# Do peripheral neuropathies differ among immune checkpoint inhibitors? Reports from the European post-marketing surveillance database in the past 10 years

**DOI:** 10.3389/fimmu.2023.1134436

**Published:** 2023-03-16

**Authors:** Rosanna Ruggiero, Nunzia Balzano, Raffaella Di Napoli, Federica Fraenza, Ciro Pentella, Consiglia Riccardi, Maria Donniacuo, Marina Tesorone, Romano Danesi, Marzia Del Re, Francesco Rossi, Annalisa Capuano

**Affiliations:** ^1^ Campania Regional Centre for Pharmacovigilance and Pharmacoepidemiology, Naples, Italy; ^2^ Department of Experimental Medicine – Section of Pharmacology “L. Donatelli”, University of Campania “L. Vanvitelli”, Naples, Italy; ^3^ Local Health Services “Napoli 1 Centro”, Naples, Italy; ^4^ Clinical Pharmacology and Pharmacogenetics Unit, Department of Clinical and Experimental Medicine, University Hospital of Pisa, Pisa, Italy

**Keywords:** immune checkpoint inhibitors, neurological toxicity, immune-related adverse events, immunotherapy, post-marketing surveillance, translational research, peripheral neuropathies

## Abstract

Although the immunotherapy advent has revolutionized cancer treatment, it, unfortunately, does not spare cancer patients from possible immune-related adverse events (irAEs), which can also involve the peripheral nervous system. Immune checkpoint inhibitors (ICIs), blocking cytotoxic T-lymphocyteassociated protein 4 (CTLA-4), programmed cell death protein 1 (PD-1), or programmed cell death ligand 1 (PD-L1), can induce an immune imbalance and cause different peripheral neuropathies (PNs). Considering the wide range of PNs and their high impact on the safety and quality of life for cancer patients and the availability of large post-marketing surveillance databases, we chose to analyze the characteristics of ICI-related PNs reported as suspected drug reactions from 2010 to 2020 in the European real-world context. We analyzed data collected in the European pharmacovigilance database, Eudravigilance, and conducted a systematic and disproportionality analysis. In our study, we found 735 reports describing 766 PNs occurred in patients treated with ICIs. These PNs included Guillain-Barré syndrome, Miller-Fisher syndrome, neuritis, and chronic inflammatory demyelinating polyradiculoneuropathy. These ADRs were often serious, resulting in patient disability or hospitalization. Moreover, our disproportionality analysis revealed an increased reporting frequency of PNs with tezolizumab compared to other ICIs. Guillain-Barré syndrome is a notable potential PN related to ICIs, as it is associated with a significant impact on patient safety and has had unfavorable outcomes, including a fatal one. Continued monitoring of the safety profile of ICIs in real-life settings is necessary, especially considering the increased frequency of PNs associated with atezolizumab compared with other ICIs.

## 1 Introduction

Peripheral neuropathies (PN) include several pathologic conditions that affect the peripheral nervous system and result from damage to peripheral nerves. Nerve injury can cause a wide range of disorders, mainly characterized by various degrees of alterations in sensitivity, pain, muscle strength and endurance, osteotendinous reflexes, and/or fine motor skills (1).

The symptoms and signs of PNs depend on the type of damaged nerves. Even if the hands and the feet are most frequently involved in PNs, the gastrointestinal, cardiovascular, and urogenital systems can also be affected (2). In particular, bowel, bladder, or digestive problems, in addition to drops in blood pressure causing dizziness, are other possible signs and symptoms of PN related to damage to autonomic nerves. So, these conditions can have a significant impact on the quality of life of patients. PNs causes can be different, like inherited and hereditary aspects, traumatic injuries, infections, metabolic problems, nutritional deficiencies, or toxic exposure, but also drugs or specific diseases can induce them (2–8). Sensorimotor polyneuropathies are often experienced by cancer patients (9), and these can be related to their oncologic condition, due to tumor invasion or compression exercised on nerves, or can be associated with a paraneoplastic effect (10). Furthermore, PNs can often also be iatrogenic, being associated with treatments (11). PNs are well-known as adverse events of classic chemotherapy or radiotherapy, which can damage healthy nerve tissue (12). Uncommonly, neuropathies can also be immune-mediated. The different possible causes make a correct differential diagnosis difficult (13).

**Figure 1:**
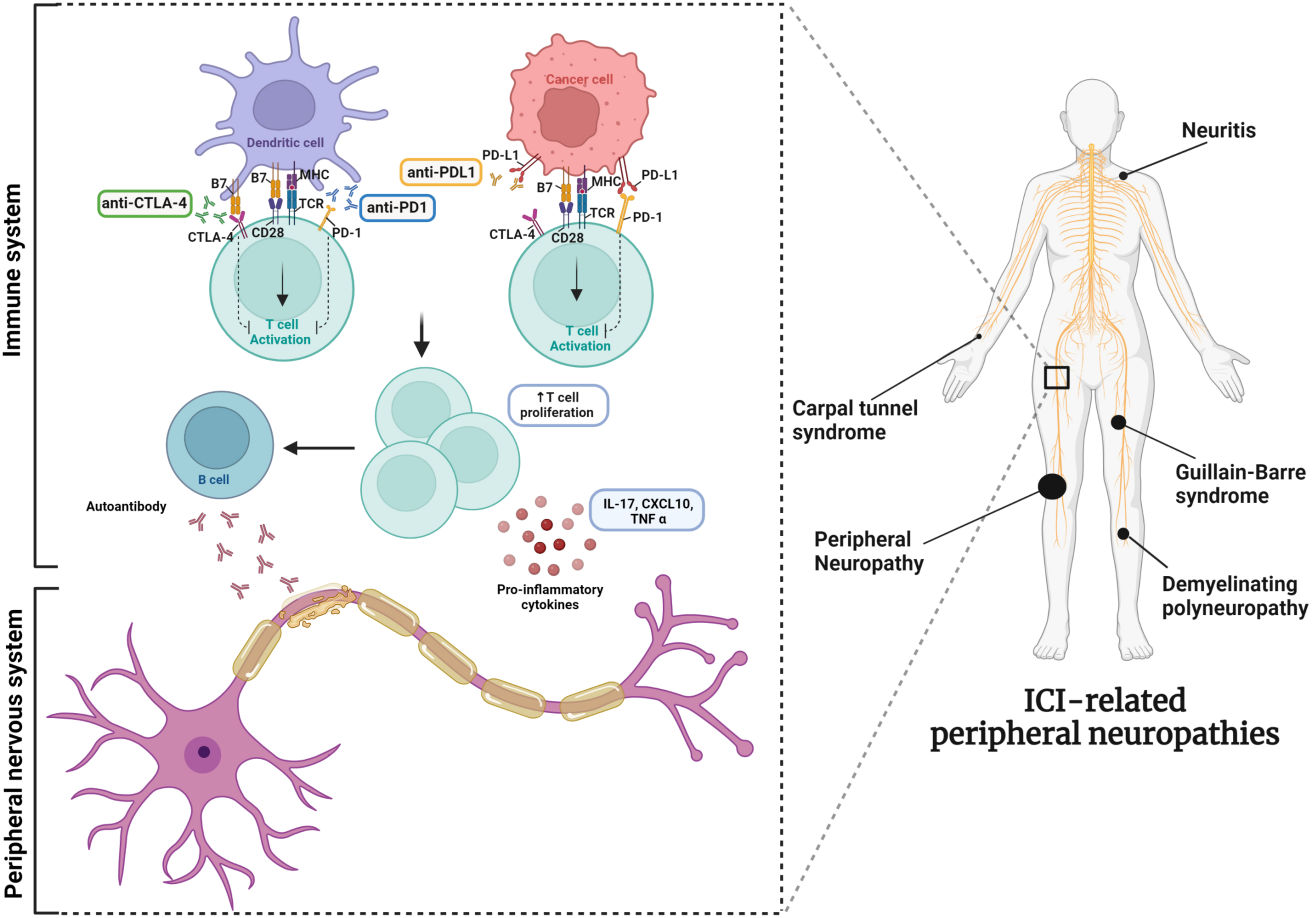
The mechanism of action of ICIs and possible associated neuropathies.

Among immune-mediated PNs, those induced by immunotherapies have recently emerged. Although the advent of oncological immunotherapy has revolutionized cancer treatment, leading to long-lasting tumor responses, it, unfortunately, does not spare cancer patients from possible adverse events, like PNs. Indeed, this new type of immune-driven neuropathy is added to the toxic neuropathies associated with exposure to traditional chemotherapies (13–15). Immune checkpoint inhibitors (ICIs), which block cytotoxic T-lymphocyte-associated protein 4 (CTLA-4), programmed cell death protein 1 (PD-1), or programmed cell death ligand 1 (PD-L1), can also induce immune imbalance and cause different immune-related adverse events (16), also in the peripheral nervous system (**Figure 1**) (17–22). From our previous study, PNs emerged as the second nervous subclass of ICI-related neurological complications more frequently described in the European pharmacovigilance database (23). Therefore, given the wide range of PNs and their impact on patients’ quality of life and the availability of large pharmacovigilance databases (24), we decided to analyze in more detail the characteristics of ICI related PNs emerging from 10 years of ICI-use in a real-world context.

## 2 Methods

### 2.1 Data sources

Large and international pharmacovigilance databases, such as the European one, Eudravigilance (EV), are important sources of clinical data, even the rarest ones. The analysis of ADR cases collected in these databases allows for the extraction of important information useful for clinicians’ prompt differential diagnosis and management. Analysis of these databases allows for constant drug safety surveillance in order to identify any new important safety signal coming from the real-world context. Therefore, for our analysis, we retrieved from the EV database all safety cases reporting PN complications that occurred in patients treated with at least one ICI and were collected in EV from 01/01/2010 until 07/02/2020. The ICIs included in our analysis were the ones authorized by the European Medicines Agency (EMA) until January 2020: ipilimumab, nivolumab, pembrolizumab, cemiplimab, atezolizumab, durvalumab, and avelumab, in mono- or combination therapies.

The EV is the European pharmacovigilance database, managed by the EMA. It collects all individual case safety reports (ICSRs) describing cases of suspected ADR related to drugs or vaccines. In fact, there are different levels of access to the data *via* the EMA website (www.adrreports.eu), the most comprehensive of which requires specific authorization from the agency. According to recent pharmacovigilance legislation, ICSR present in the EV database can be reported both by a healthcare professional and a non-healthcare professional (e.g., a citizen or other professional figures). The reported adverse events are categorized according to the Medical Dictionary for Regulatory Activities (MedDRA). According to a hierarchical structure, this dictionary is characterized by five different levels, from the lowest one (the lowest level term, LLT), up to the highest one (the System Organ Class, SOC). Thus, it is possible to select specific cases in EV by searching for SOCs. In light of this, we selected all cases reporting at least one ICI as a suspected drug and an ADR belonging to the SOC “Neurological disorders”, focusing our analysis on those cases that described at least one neurological complication belonging to the MedDRA “Peripheral neuropathies” High-Level Group Terms (HLGT).

### 2.2 Descriptive analysis

ADR reports were analyzed for patient characteristics, describing age group, gender, and therapies, and differentiating suspected ICI (ipilimumab, nivolumab, pembrolizumab, cemiplimab, atezolizumab, durvalumab, avelumab, or combination ICI treatments) from other suspected or concomitant drugs (Level II ATC). Moreover, reports were categorized by source, including reporter type (Healthcare Professional, HCP, or Non-Healthcare Professional, N-HCP), and the country for regulatory purposes, distinguished into the European Economic Area and the Non-European Economic Area. Our aim was to compare which type of PN was more frequently reported as a suspected ADR for each ICI treatment. PNs were described in terms of action taken to manage them, outcome, and severity, with severity criteria specified in accordance with the International Council on Harmonization E2D guidelines. In particular, an ADR was considered serious when it resulted in death, hospitalization or its prolongation, severe or permanent disability, or congenital anomalies/birth defects, or if it was a life-threatening or clinically relevant event. The outcome of neurological complications was classified as “recovered/resolved”, “recovering/resolving”, “recovered/resolved with sequelae”, “not recovered/not resolved”, “fatal”, and “unknown”. Moreover, PNs were categorized according to the MedDRA High-Level Terms (HLT), and the different neurological diagnoses (p-term) included in each reference HLT.

### 2.3 Disproportionality analysis

In order to compare the frequency of PNs reporting between each ICI or ICI class, we performed] disproportionality analysis applying the Reporting Odds Ratio (ROR) of 95% CI and considered a statistically significant signal if the lower limit of the 95% CI of a ROR exceeded 1.0. For our analysis, we considered the first and most frequent PNs belonging to each different HLT.

Thus, RORs were calculated by comparing each ICI treatment (ipilimumab, nivolumab, pembrolizumab, atezolizumab, durvalumab, ipilimumab/nivolumab) with each other. We also classified ICIs based on their mechanism of action into anti-CTLA-4 (ipilimumab), anti-PD-1 (nivolumab and pembrolizumab), and anti-PDL-1 (atezolizumab and durvalumab). Therefore, ROR was also performed comparing ICI classes using other classes as comparators (anti-CTLA-4 *vs* anti-PD-1 or anti-PDL-1 and anti-PD-1 *vs* anti-PDL-1). The Rstudio software was used to perform the disproportionality analysis.

## 3 Results

### 3.1 Descriptive analysis

Overall, we found 735 ICSRs that reported at least one ICI as a suspected drug and described PNs as ADRs in EV. Safety reports were mainly related to nivolumab, followed by pembrolizumab and ipilimumab. Specifically, 264 (35.9%) ICSRs were related to nivolumab, 193 (26.3%) to pembrolizumab, and 109 (14.8%) to ipilimumab. The nivolumab/ipilimumab association was reported as a suspected drug in 89 (12.1%) ICSRs. Other ICIs were reported less frequently (<10%), of which cemiplimab was the least reported, resulting in only 1 (0.1%) ICSR (**Table 1**). The majority of reports were in elderly patients (N=340; 46.3%) and reported by HCPs (N=682; 92.8%). Among the more frequently reported ICIs (> 100 ICSRs), elderly patients were particularly represented in nivolumab-related ICSRs (N=137; 51.9%) (**Table 1**).

**Table 1:** Demographic characteristics and distribution for primary source, country of primary source, number of suspected drugs other than immune checkpoint inhibitors (ICIs), and number of concomitant drugs of Individual Case Safety Reports (ICSRs) reporting at least one peripheral nerve adverse event and having at least one ICI as suspected drug. Data collected in Eudravigilance from the date of each ICI marketing authorization up to 07/02/2020.

Variable	Level	All ICSRs* (N=735; 100%)	ICSRs with nivolumab (N=264; 35.9%)	ICSRs with pembrolizumab (N=193; 26.3%)	ICSRs with ipilimumab (N=109; 14.8%)	ICSRs with durvalumab (N=17; 2.3%)	ICSRs with atezolizumab (N=51; 6.9%)	ICSRs with nivolumab and ipilimumab (N=89; 12.1%)
***Age Group***	*Adult*	269 (36.6)	95 (36.0)	65 (33.7)	39 (35.8)	7 (41.2)	26 (51.0)	34 (38.2)
	*Elderly*	340 (46.3)	137 (51.9)	92 (47.7)	50 (45.9)	7 (41.2)	16 (31.4)	32 (36.0)
	*Not specified*	126 (17.1)	32 (12.1)	36 (18.6)	20 (18.3)	3 (17.6)	9 (17.6)	23 (25.8)
***Gender***	*W (%)*	235 (32.0)	76 (28.8)	68 (35.2)	27 (24.8)	7 (41.2)	20 (39.2)	31 (34.8)
	*M (%)*	458 (62.3)	175 (66.3)	108 (56.0)	75 (68.8)	9 (52.9)	30 (58.8)	55 (61.8)
	*Missing (%)*	42 (5.7)	13 (4.9)	17 (8.8)	7 (6.4)	1 (5.9)	1 (2.0)	3 (3.4)
***Primary Source***	*Healthcare Professional*	682 (92.8)	243 (92.0)	187 (97.0)	93 (85.3)	16 (94.1)	46 (90.2)	85 (95.5)
	*Non-Healthcare Professional*	52 (7.1)	20 (7.6)	6 (3.0)	16 (14.7)	1 (5.9)	5 (9.8)	4 (4.5)
	*Not available*	1 (0.1)	1 (0.4)	–	–	–	–	–
***Primary Source Country for Regulatory Purposes***	*European Economic Area*	271 (36.9)	111 (42.0)	55 (28.5)	43 (39.4)	7 (41.2)	14 (27.5)	37 (41.6)
	*Non-European Economic Area*	463 (63.0)	152 (57.6)	138 (71.5)	66 (60.6)	10 (58.8)	37 (72.5)	52 (58.4)
	*Not available*	1 (0.1)	1 (0.4)	–	–	–	–	–
***Suspected drug(s) other than ICIs***	*0*	604 (82.2)	224 (85.0)	151 (78.2)	98 (90.0)	11 (64.7)	29 (56.9)	81 (91.0)
	*1*	51 (6.9)	17 (6.4)	16 (8.5)	5 (4.6)	2 (11.8)	8 (15.7)	3 (3.4)
	*2*	49 (6.7)	15 (5.7)	21 (10.9)	5 (4.6)	2 (11.8)	3 (5.8)	2 (2.2)
	*3*	20 (2.7)	4 (1.5)	2 (1.0)	1 (0.8)	–	11 (21.6)	1 (1.1)
	*4*	4 (0.5)	1 (0.4)	1 (0.5)	–	2 (11.8)	–	–
	≥ 5	7 (1.0)	3 (1.0)	2 (1.0)	–	–	–	2 (2.3)
***Concomitant drug(s)***	*0*	521 (70.9)	197 (74.7)	128 (66.3)	72 (66.0)	9 (52.9)	38 (74.5)	72 (80.9)
	*1*	42 (5.7)	12 (4.5)	18 (9.3)	5 (4.6)	–	3 (5.9)	3 (3.4)
	*2*	39 (5.3)	9 (3.4)	10 (5.2)	6 (5.5)	3 (17.6)	4 (7.8)	5 (5.6)
	*3*	26 (3.5)	6 (2.3)	9 (4.6)	6 (5.5)	–	1 (2.0)	3 (3.4)
	*4*	14 (1.9)	3 (1.1)	3 (1.6)	3 (2.8)	1 (6.0)	1 (2.0)	2 (2.2)
	≥ 5	93 (12.7)	37 (14.0)	25 (13.0)	17 (15.6)	4 (23.5)	4 (7.8)	4 (4.5)

*The ICSRs for cemiplimab, avelumab, and other combination therapies are shown in the **Supplementary Material (Table S1)**. "-" means that 0 cases were reported.

The reporter distribution remained the same for each ICI, for which more than 80% of ICSRs were reported by HCPs. Ipilimumab-related ICSRs showed a slightly higher percentage of N-HCPs (14.7%) as the reported source compared to the other ICIs (**Table 1**).

Looking at all ICSRs, the male gender was more frequently represented (N=458; 62.3%). Similarly, when our dataset was also stratified for each suspected drug ICI, the male gender was reported in more than 50% of ICSRs, particularly for those related to ipilimumab (68.8%) or nivolumab (66.3%). Only in four ICSRs related to the association of ipilimumab/pembrolizumab, the female gender was represented in 75.0% of ICSRs (**Table 1**).

In terms of the primary country of origin for regulatory purposes, the Non-European Economic Area was the most representative one for all ICSRs (N=463; 63.0%), especially for those related to atezolizumab (37 out of 51; 72.5%) and pembrolizumab (138 out of 193; 71.5%) (**Table 1**).

As more than one ADR can be reported in each ICSR, we overall observed a total of 766 neurological irADRs categorized as PN according to MedDRA (**Table 2**). Among these, the adverse events were more frequently reported with a generic term, such as “peripheral neuropathy” (N=335; 43.7%) and “polyneuropathy” (N=69; 9.0%) p-terms. Specific syndromes were also reported, such as Guillain-Barré syndrome (GBS) (N=154; 20.1%), carpal tunnel syndrome (N=13; 1.7%), and Miller-Fisher syndrome (N=4; 0.5%) (**Table 2**). Regarding the gender distribution of the reported PNs, men continued to be more commonly involved in the reported of GBS (M =477; 62.0% *vs* F=247; 32.2%). In contrast, carpal tunnel syndrome (F=9; 1.2% vs M=4; 0.5%) and Miller-Fisher syndrome (F=2; 1.2% vs M=1; 0.5%) were more frequently reported in women.

**Table 2:** Distribution of neurological complications included in the “Peripheral neuropathies” HGLT that occurred in European patients treated with at least one ICI.

High-Level Terms	TOT* (N=766)	Ipilimumab (N=114)	Nivolumab (N=272)	Pembrolizumab (N=203)	Atezolizumab (N=52)	Durvalumab (N=17)	Ipilimumab/nivolumab (N=94)
**Peripheral neuropathies (NEC)**	**496 (64.8)**	**70 (61.4)**	**182 (66.9)**	**133 (65.7)**	**38 (73.1)**	**14 (82.4)**	**51 (54.2)**
Peripheral neuropathy	335 (43.7)	49 (43.0)	120 (44.1)	90 (44.3)	33 (63.5)	8 (47.1)	30 (31.9)
Polyneuropathy	69 (9.0)	10 (8.8)	24 (8.8)	16 (7.9)	4 (7.7)	2 (11.8)	11 (11.7)
Peripheral sensory neuropathy	42 (5.5)	5 (4.4)	17 (6.3)	15 (7.4)	1 (1.9)	3 (17.6)	1 (1.1)
Autoimmune neuropathy	18 (2.3)	2 (1.8)	6 (2.2)	4 (2.0)	-	1 (5.9)	5 (5.3)
Peripheral motor neuropathy	10 (1.3)		5 (1.8)	4 (2.0)	-	-	1 (1.1)
Axonal neuropathy	4 (0.5)	–	–	3 (1.5)	–	–	-
Peripheral sensorimotor neuropathy	7 (0.9)	3 (2.6)	2 (0.7)	–	–	–	2 (2.1)
Brachial plexopathy	3 (0.4)	–	2 (0.7)	–	–	–	1 (1.1)
Immune-mediated neuropathy	3 (0.4)	–	2 (0.7)	1 (0.5)	–	–	–
Toxic neuropathy	3 (0.4)	1 (0.9)	2 (0.7)	–	–	–	–
Neuralgic amyotrophy	2 (0.3)	-	2 (0.7)	–	–	–	–
**Acute polyneuropathies**	**165 (21.5)**	**31 (27.2)**	**49 (18.0)**	**45 (22.0)**	**7 (13.6)**	**2 (11.8)**	**26 (27.6)**
Guillain- Barré syndrome	154 (20.1)	29 (25.4)	46 (16.9)	41 (20.2)	7 (13.5)	2 (11.8)	25 (26.5)
Acute polyneuropathy	7 (0.8)	1 (0.9)	2 (0.7)	2 (1.0)	–		1 (1.1)
Acute motor axonal neuropathy	2 (0.3)	-	1 (0.4	1 (0.5)	–	–	–
Acute motor-sensory axonal neuropathy	2 (0.3)	1 (0.9)	–	1 (0.5)	–	–	–
**Chronic polyneuropathies**	**45 (5.9)**	**8 (7.0)**	**17 (6.2)**	**9 (4.4)**	**2 (3.8)**	**-**	**8 (8.5)**
Demyelinating polyneuropathy	29 (3.8)	6 (5.3)	11 (4.0)	5 (2.5)	2 (3.8)	–	5 (5.3)
Chronic inflammatory demyelinating polyradiculoneuropathy	12 (1.6)	2 (1.8)	4 (1.5)	3 (1.5)	–	–	2 (2.1)
Diabetic neuropathy	2 (0.3)	-	1 (0.4)	1 (0.5)	–	–	
Multifocal motor neuropathy	1 (0.1)	–	–	–	–	–	1 (1.1)
Polyneuropathy in malignant diseases	1 (0.1)	-	1 (0.4)	–	–	–	–
**Mononeuropathies**	**37 (4.8)**	**4 (3.5)**	**15 (5.5)**	**11 (5.5)**	**3 (5.7)**	**-**	**4 (4.3)**
Carpal tunnel syndrome	13 (1.7)	1 (0.9)	7 (2.6)	5 (2.5)	–	–	–
Mononeuropathy multiplex	6 (0.8)	-	5 (1.8)	1 (0.5)	–	–	–
Peroneal nerve palsy	5 (0.7)	1 (0.9)	–	1 (0.5)	1 (1.9)	–	2 (2.1)
Phrenic nerve paralysis	5 (0.7)	2 (1.8)	–	1 (0.5)	1 (1.9)	–	1 (1.1)
Mononeuritis	3 (0.4)	–	–	2 (1.0)		–	1 (1.1)
Mononeuropathy	1 (0.1)	–	–	-	1 (1.9)	–	–
Peripheral nerve lesion	1 (0.1)	–	–	1 (0.5)	–	–	–
Peripheral nerve palsy	1 (0.1)	–	1 (0.4)	–	–	–	–
Radial nerve palsy	1 (0.1)	–	1 (0.4)	–	–	–	–
Sciatic nerve neuropathy	1 (0.1)	–	1 (0.4)	–	–	–	–
**Peripheral neuropathies**	**19 (2.5)**	**1 (0.9)**	**9 (3.3)**	**4 (1.9)**	**1 (1.9)**	**-**	**4 (4.3)**
Neuritis	19 (2.5)	1 (0.9)	9 (3.3)	4 (1.9)	1 (1.9)	–	4 (4.3)
**Acute peripheral neuropathies**	**4 (0.5)**	–	–	**1 (0.5)**	**1 (1.9)**	**1 (5.8)**	**1 (1.1)**
Miller-Fisher syndrome	4 (0.5)	–	–	1 (0.5)	1 (1.9)	1 (5.8)	1 (1.1)

*Neurological events related to cemiplimab, avelumab, and other combination therapies are shown in the **Supplementary Material (Table S2)**.

Neurological complications were categorized according to MedDRA High-Level Terms (HLT) and the ICI treatments involved. The bold values are referred to High level terms, while the no-bold values are referred to Prefered term included in the reference High Level Term. "-" means that 0 cases were reported.

We categorized all reported PNs, such as ICI-related irADRs, according to the MedDRA HLTs in **Table 2**. “Peripheral neuropathies NEC” (N=496; 64.8%) were the most common HLT.

Overall, we considered 32 different disorders or clinical diagnoses grouped into six different HLTs, such as the categories of acute (N=165; 21.5%) or chronic polyneuropathies (N=45; 5.9%), and mononeuropathies (N=37; 4.8%). GBS was the most frequently reported PN among acute polyneuropathies (N=154; 20.1%). In particular, GBS was mainly reported in ICSRs related to nivolumab (N=46) and pembrolizumab (N=41). Among the chronic polyneuropathies, CIDP was described in 12 cases, mainly related to nivolumab (N=4) and pembrolizumab (N=3), while only one case of MMN was reported, as ADRs occurred in patients treated with the ipilimumab/nivolumab combination. Moreover, 29 cases of demyelinating polyneuropathy (3.8%), mainly related to nivolumab (N=11), and two cases of diabetic neuropathy (0.3%) were also described. The latter were reported as suspected ADRs of nivolumab (N=1) and pembrolizumab (N=1).

As reported in **Table 2**, the majority of events were related to anti-PD1 agents, nivolumab (N=272; 35.5%) and pembrolizumab (N=203; 26.5%), followed by the anti-CTLA4 drug ipilimumab (N=114; 14.9%), the combination therapy ipilimumab/nivolumab (N=94; 12.3%), and atezolizumab (N=52; 7.0%). Approximately 2.2% of PNs were attributed to durvalumab (N=17). We found only one case related to cemiplimab. This latter described a case of polyneuropathy occurred in an elderly male patient treated with cemiplimab in off-label use. Nevertheless, the therapeutic indication for cemiplimab was expressed generically as malignant neoplasm.

In the majority of ICSRs, the reported PNs resulted in serious ADRs (95.2%), most of which were medically significant (N=335; 43.7%), or caused or prolonged hospitalization (N=228; 29.8%). Moreover, PNs resulted in death in 81 cases (10.6%) or disability in 43 cases (5.6%) (**Table 3**). In particular, the higher percentages of disabling PNs were associated with pembrolizumab (N=15; 7.4%) and ipilimumab (N=8; 7.0%). In the same table, we reported the distribution of PNs by outcome. The outcome was unknown in 45.5% of cases, especially for PNs related to ipilimumab therapy (55.3%). Overall, 22.2% of ICI-related PNs had unfavorable outcomes, defined as “not resolved” or “resolved with sequelae” at the time of reporting (20.9% and 1.3%, respectively). Positive outcomes were reported in 28.5% of adverse events. In particular, 9.7% of PNs (N=74) were completely resolved, while 18.8% (N=144) improved. In contrast, a fatal outcome was observed in 3.8% (N=29) and Guillain-Barré syndrome was the most common PN (N=12; 41.4%) among those with fatal outcomes.

**Table 3:** Distribution of severity and outcomes of neurological complications occurred in European patients treated with at least one ICI.

	Number of neurological events TOTAL (n=766)	Number of events with nivolumab (n=272)	Number of events with pembrolizumab (n=203)	Number of events with ipilimumab (n=114)	Number of events with durvalumab (n=17)	Number of events with atezolizumab (n=52)	Number of events with avelumab (n=3)	Number of events with cemiplimab (n=1)	Number of events with two or more ICIs (n=104)
**Severity**									
Not available	37 (4.8)	15 (5.5)	7 (3.4)	5 (4.4)	3 (17.6)	4 (7.7)	–	–	3 (2.9)
Severe	729 (95.2)	257 (94.5)	196 (96.6)	109 (95.6)	14 (82.4)	48 (92.3)	–	–	1 (97.1)
**Severity Criteria**									
Other medically important conditions	335 (43.7)	127 (46.7)	79 (38.9)	53 (46.5)	5 (29.4)	20 (38.5)	1 (33.3)	1 (100)	49 (47.1)
Results in death	81 (10.6)	39 (14.3)	20 (9.9)	15 (13.2)	–	3 (5.8)	–	–	4 (3.8)
Caused/prolonged hospitalization	228 (29.8)	69 (25.4)	67 (33.0)	29 (25.4)	7 (41.2)	21 (40.4)	–	–	35 (33.7)
Life-threatening	42 (5.5)	8 (3.0)	15 (7.4)	4 (3.5)	1 (5.9)	3 (5.8)	2 (66.7)	–	9 (8.7)
Disability	43 (5.6)	14 (5.1)	15 (7.4)	8 (7.0)	1 (5.9)	1 (1.8)	–	–	4 (3.8)
**Outcome**									
Recovered/resolved	74 (9.7)	29 (10.7)	17 (8.4)	11 (9.6)	3 (17.6)	4 (7.7)	–	–	10 (9.6)
Recovering/resolving	144 (18.8)	59 (21.7)	35 (17.2)	14 (12.3)	2 (11.8)	9 (17.3)	–	–	25 (24.0)
Recovered/resolved with sequelae	10 (1.3)	4 (1.5)	3 (1.5)	2 (1.7)	–	–	1 (33.3)	–	–
Not recovered/Not resolved	160 (20.9)	47 (17.3)	62 (30.5)	18 (15.8)	4 (23.5)	14 (26.9)	–	–	15 (14.4)
Fatal	29 (3.8)	17 (6.2)	5 (2.5)	6 (5.3)	–	–	–	–	1 (1.0)
Unknown	349 (45.5)	116 (42.6)	81 (39.9)	63 (55.3)	8 (47.1)	25 (48.1)	2 (6.7)	1 (100)	53 (51.0)

"-" means that 0 cases were reported.

In 131 ICSRs, suspected drugs other than ICIs were reported (**Table 1**); in the majority of cases, only one (N=51) or two (N=49) suspected drugs other than ICIs were reported. Looking at each ICI, suspect drugs were reported in 40 of 264 nivolumab-related ICSRs (15.2%), 42 of 193 pembrolizumab-related ICSRs (21.8%), 11 out of 109 ipilimumab-related ICSRs (10.1%), 22 of 51 atezolizumab-related ICSRs (43.1%), six of 17 durvalumab- related ICSRs (35.3%) and one of 2 avelumab-related ICSRs (50.0%) (**Table 1**). The most frequently reported other suspect drugs were other antineoplastic agents (N=206; 73.3%), in particular taxanes (N=56) and platinum compounds (N=51). The distribution of other suspected drugs for Level II ATC is reported in **Table 4**.

**Table 4:** Drugs reported as suspected non-ICIs in Individual Case Safety Reports (ICSRs) collected in Eudravigilance, categorized by therapeutic class (Level II ATC).

ATC CODE	ATC NAME	N	%
**L01**	Antineoplastic agents	206	73.3%
**A07**	Antidiarrheals, intestinal anti-inflammatory/anti-infective agents	6	2.1%
**D07**	Corticosteroids, dermatological preparations	6	2.1%
**J05**	Antivirals for systemic use	4	1.4%
**N02**	Analgesics	4	1.4%
**B01**	Antithrombotic Agents	3	1.1%
**J07**	Vaccines	3	1.1%
**L04**	Immunosuppressants	3	1.1%
**N01**	Anesthetics	3	1.1%
**N05**	Psycholeptics	3	1.1%
**A01**	Stomatological Preparations	2	0.7%
**A02**	Antacids	2	0.7%
**A03**	Combinations of psycholeptics and antispasmodics	2	0.7%
**A06**	Drugs for Constipation	2	0.7%
**A10**	Drugs used in diabetes	2	0.7%
**A11**	Vitamins	2	0.7%
**B02**	Antihemorrhagics	2	0.7%
**C01**	Cardiac therapy	2	0.7%
**C10**	Lipid-modifying agents	2	0.7%
**H01**	Pituitary and hypothalamic hormones and analogs	2	0.7%
**J01**	Antibacterials for systemic use	2	0.7%
**L03**	Immunostimulants	2	0.7%
**M02**	Topical products for joint and muscular pain	2	0.7%
**M05**	Drugs for the treatment of bone diseases	2	0.7%
**R03**	Drugs for obstructive airway diseases	2	0.7%
**V03**	All other therapeutic products	2	0.7%
**B03**	Anti-anemic preparations	1	0.4%
**B05**	Blood substitutes and perfusion solutions	1	0.4%
**C07**	Beta blocking agents	1	0.4%
**J06**	Immune sera and immunoglobulins	1	0.4%
**N03**	Antiepileptic drugs	1	0.4%
**R01**	Nasal preparations	1	0.4%
**R05**	Cough and cold preparations	1	0.4%
**S02**	Otologicals	1	0.4%

**Figure 2:**
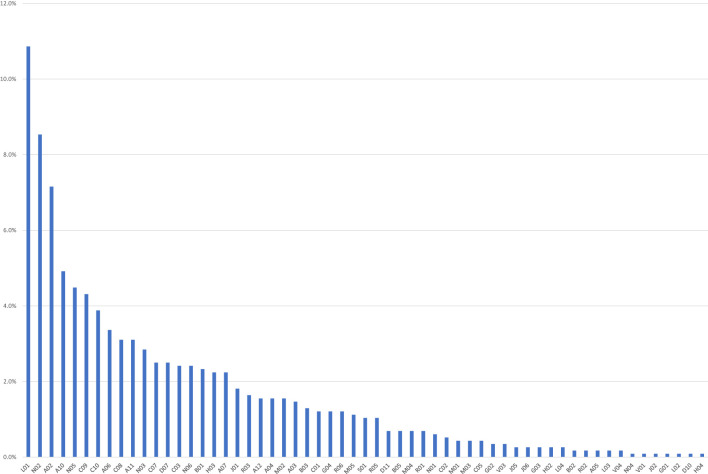
Drugs reported as concomitants in the Individual Case Safety Reports (ICSRs) collected in Eudravigilance, categorized by therapeutic reference class (Level II ATC). A02, Antacids; A03, Combinations of psycholeptics and antispasmodics; A04, Antiemetics and antinausea; A05, Biliary and hepatic therapy; A06, Drugs for constipation; A07, Antidiarrheals, anti-inflammatory/anti-infective agents; A10, Drugs used in diabetes; A11, Vitamins; A12, Mineral supplements; B01, Antithrombotic agents; B02, Antihemorrhagics; B03, Antianemic preparations; B05, Blood substitutes and perfusion solutions; C01, Cardiac therapy; C02, Antihypertensives; C03, Diuretics; C05, Vasoprotectives; C07, Beta blocking agents; C08, Calcium channel blockers; C09, Agents acting on the renin–angiotensin; C10, Lipid modifying agents; D07, Corticosteroids. dermatological preparations; D10, Anti-acne preparations; D11, Other dermatological preparations; G01, Gynecological anti-infectives and antiseptics; G02, Other gynecologicals; G03, Sex hormones and modulators of the genital system; G04, Urologicals; H02, Corticosteroids for systemic use; H03, Thyroid therapy; H04, Pancreatic hormones; J01, Antibacterials for systemic use; J02, Antimycotics for systemic use; J05, Antivirals for systemic use; J06, Immune sera and immunoglobulins; L01, Antineoplastic agents; L02, Endocrine therapy; L03, Immunostimulants; L04, Immunosuppressants; M01, Anti-inflammatory and antirheumatic products; M02, Topical products for joint and muscular pain; M03, Muscle relaxants; M04, Antigout preparations; M05, Drugs for treatment of bone diseases; N01, Anesthetics; N02, Analgesics; N03, Antiepileptics; N04, Anti-Parkinson drugs; N05, Psycholeptics; N06, Psychoanaleptics; R01, Nasal preparations; R02, Throat preparations; R03, Drugs for obstructive airway diseases; R05, Cough and cold preparations; R06, Antihistamines for systemic use; S01, Ophthalmologicals; V01, Allergens; V03, All other therapeutic products; V04, Diagnostic agents. Six drugs (0.5%) reported as concomitant were officinal drugs.

In the majority of ICSRs (N=521; 70.9%), there were no other concomitant medications, which were only reported in 214 ICSRs. More than five concomitant drugs were reported in 93 ICSRs, mainly related to nivolumab (N=37), followed by pembrolizumab (N=25). Generally, the most frequently reported concomitant drug classes were other antineoplastic agents (N=126; 10.9%), followed by analgesics (N=99; 8.5%) and drugs for acid-related disorders (N=83; 7.2%). The distribution of concomitant drugs for Level II ATC is reported in **Figure 2**.

Finally, when considering the reported actions taken to manage the adverse events, drug withdrawal was reported in 53.6% of cases (N=394), while the suspected ICI dose remained unchanged in 7.2% of cases (N=53). Only one report described a peripheral neuropathy managed with the ICI dosage reduction. This case was referred to an adult male patient (age group 18-64 years) affected by Hodgkin's disease in treatment with nivolumab (3 mg/kg/iv).

### 3.2 Disproportionality analysis

**Figure 3:**
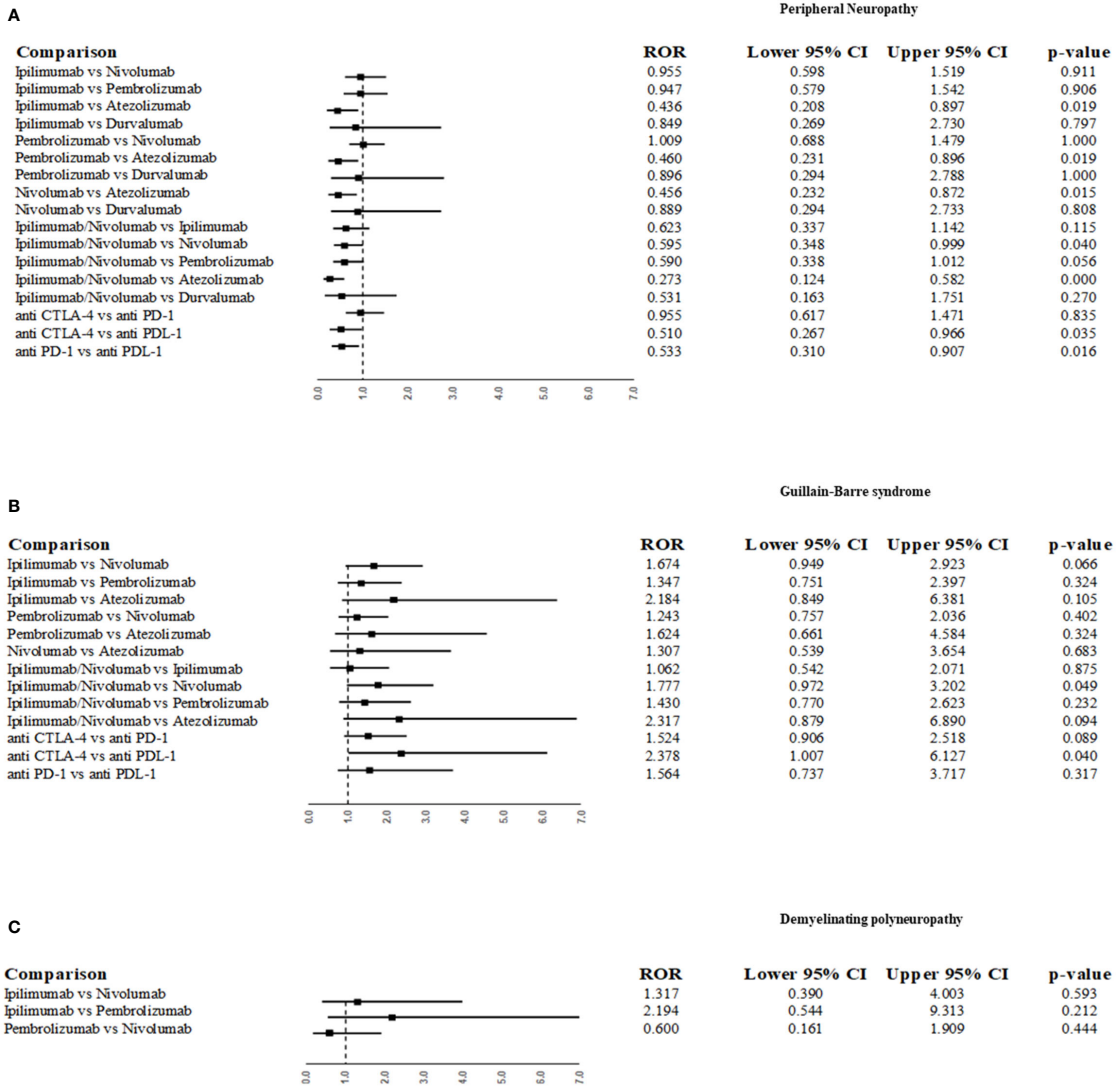
Reporting odds ratio (ROR) for disproportionality analysis of the most frequently reported peripheral neuropathies as suspected ADRs associated with ICIs. ROR was performed for the following p-term “neuropathy peripheral” **(A)**, “Guillain-Barre syndrome” **(B)** and “demyelinating polyneuropathy” **(C)**.

The most common PNs belonging to the “Peripheral neuropathies NEC”, “Acute polyneuropathies” and “Chronic polyneuropathies” HLTs were “peripheral neuropathy”, “Guillain-Barré syndrome” and “demyelinating polyneuropathy”, respectively (**Table 2**). We, therefore, applied ROR to these adverse events by comparing different ICIs or ICI classes between them. As reported in section A of **Figure 3**, ipilimumab, pembrolizumab, and nivolumab were associated with a lower reporting probability of “peripheral neuropathy” (p-term) compared to atezolizumab (ROR 0.436; 95% CI 0.208-0.897; p=0.019; ROR 0.46; 95% CI 0.231-0.896; p=0.019; ROR 0.456; 95% CI 0.232-0.872; p=0.015, respectively). The ipilimumab/nivolumab association also showed a lower probability of reporting peripheral neuropathy compared to atezolizumab (ROR 0.3, 95% CI 0.1-0.6; p <0.001). Moreover, when comparing ICI classes, anti-CTLA-4 and anti-PD-1 classes were associated with a lower likelihood of reporting peripheral neuropathy compared to anti-PDL-1 (ROR 0.510; 95% CI 0.267-0.966; p=0.03; ROR 0.533; 95% CI 0.31-0.907; p=0.016, respectively). No significant statistical difference was observed when comparing ICI or ICIs classes for the reporting probability of Guillain-Barré syndrome (**Figure 3B**), nor for demyelinating polyneuropathy, except for a higher reporting probability of Guillain-Barré syndrome for anti-CTLA4 compared to PD-L1 (ROR 2.378; 95% CI 1.007-6.127;p=0.04).

## 4 Discussion

ICI-related neuropathies represent one of the most frequently reported neurological complications in the European database (23). Given how in our study the PNs described in the majority of retrieved ICSRs were categorized as serious, these adverse events represent an important safety concern for the therapeutic use of ICIs. Severe motor or sensory neuropathies are among the complications of ICIs that may require permanent discontinuation of ICI therapy. Moreover, even if ICI withdrawal may not be necessary, the occurrence of PNs can have a critical impact on the patient’s quality of life and lead to negative outcomes. Immunotherapy-related neuropathies represent a small subset of autoimmune PNs due to an aberrant immune response against components of the peripheral nervous system. This abnormal immune response induced by immunotherapeutic agents can lead to various adverse manifestations, characterized by possible demyelination processes or axonal damages (25, 26). In our dataset, both types of injuries emerged, although demyelinating PNs were more frequently reported than axonal injuries associated with ICIs. Among the demyelinating disorders, we found several cases of Guillain-Barré syndrome, demyelinating polyneuropathy, and chronic inflammatory demyelinating polyradiculoneuropathy (CIDP). We also found some cases of ICI-related Miller-Fisher syndrome, a rare variant of Guillain-Barré syndrome characterized by a triad of symptoms: ophthalmoplegia, ataxia, and areflexia (26). Fortunately, these neurologically adverse events are rare. According to a systematic review conducted by Yan Li et al., from 1990 up to 2021, only 33 ICI-related case reports of Guillain-Barré syndrome or its subtypes, such as Miller-Fisher syndrome (MFS), acute inflammatory demyelinating polyneuropathy (AIDP), and acute motor axonal neuropathy (AMAN), were described in the literature (27). In our analysis, a few cases of AMAN and motor-sensory axonal neuropathy emerged, which were mainly related to pembrolizumab. Although rare, these adverse events require special attention from clinicians given their potentially serious consequences. In addition to the characteristic damage type, PNs can also be categorized based on the time of onset and duration. According to our results, the acute forms were more frequently reported as ICI-related ADRs than the chronic ones. Among the latter, our dataset described some cases of chronic inflammatory demyelinating polyradiculoneuropathy (CIDP), an immune-mediated progressive neuropathy characterized by electrodiagnostic evidence of peripheral nerve demyelination, and only one case of multifocal motor neuropathy (MMN). According to our results, these chronic forms were mainly related to nivolumab treatment, either as monotherapy or in association with ipilimumab. According to Okada et al., polyradiculoneuropathy induced by ICIs presents specific clinical features, such as severe motor weakness affecting both legs symmetrically and resulting in gait disturbance, and an effective response to steroid treatment for its management. In addition, the authors suggested its early detection by electrodiagnostic evaluation of demyelination and cerebrospinal fluid, typically characterized by elevated lymphocytes (28). Other case reports have been described in the literature describing acute sensorimotor neuropathy and polyneuropathy occurring in patients treated with ipilimumab (25, 29). The possible relationship between the occurrence of neuropathy events and the administration of ICIs is supported by a close temporal association between symptomatic onset and a positive response to corticosteroids or immunomodulatory therapy (30). Among the acute PNs emerging from our analysis, GBS is noteworthy. In particular, it was reported in 20% of ICSRs. Moreover, Guillain-Barré syndrome was associated with a significant impact on patient safety, as it was the most prevalent PN with adverse outcomes, including fatalities. As reported in the literature, there is an increased incidence of fatal Guillain-Barré syndrome in patients treated with immune checkpoint inhibitors (31). Overall, fatal cases in our dataset were more frequently related to nivolumab and ipilimumab. Moreover, the majority of disabling PNs were also related to anti-PD-1 agents, nivolumab, and pembrolizumab. Overall, the majority of all ICSRs were related to these three ICIs, which are the older approved ICIs and therefore the most widely used and longest used in clinical practice. In the same way, the higher incidence of tumor pathologies like lung cancer, melanoma, and renal cell cancer in elderly male patients should be considered when looking at the gender and age distribution of the reported PNs. In fact, these tumors are more common in adult or elderly men. However, the data in the literature regarding the greater susceptibility of men to ICI-related irADRs, including PNs, are contradictory and still controversial, requiring further investigation (32).

Concerning the biological plausibility of ICI-related PNs, results from experimental and clinical studies confirm the possible immune-mediated pathogenesis of these disorders. In particular, inhibition of T-reg cell activity may be one potential mechanism for breaking immune tolerance. It has been shown that T-reg cells expressing PD-1 and CTLA-4 receptors on their surfaces are involved in self-tolerance processes. AntiPD-1 and antiCTLA-4 inhibitors block immune checkpoints that break physiological immune tolerance, with a consequent hyperproliferation and hyperactivation of immune effector cells, which can lead to the development of neurological adverse events, such as peripheral neuropathies (33). Humoral and/or cellular immune mechanisms against Schwann cell/myelin antigens may underlie or participate in the pathogenesis of ICI-related PNs (34, 35). This pathogenetic mechanism should be linked to the phenomenon of molecular mimicry in terms of cross-reactivity between the tumor antigens and similar epitopes on healthy peripheral nerve cells. In addition, epitope spreading (36, 37) is one of the suggested mechanisms. PNs can be stimulated to increase the production of inflammatory cytokines, such as TNF-alpha and IL-17, by activated effector T-cells and their consequent effects on the structures of the peripheral nervous system (29). As suggested by Xi Chen et al., another possible hypothesis may be related to a pre-existing neuropathy, which may be induced by previous or concurrent chemotherapy or due to comorbidities such as diabetes. A pre-existing condition could increase the patient’s susceptibility to immune-mediated ICIs complications, which could worsen after ICIs treatments (30). In our dataset, 107 ICSRs reported at least one anticancer agent as other suspect drugs, which is considered a predisposing factor or alternative cause of PNs. Si Zhihua et al. described a higher risk of developing peripheral neuropathy when PD-1/PD-L1 inhibitors were used in combination with chemotherapy (38). Similarly, Yuan Tian et al. also showed an increased incidence trend of neurological toxicities, especially grade 3–5 peripheral neuropathy, with anti-PD-1 and anti-PD-L1 plus chemotherapy (39).

Finally, our disproportionality analysis surprisingly revealed an increased frequency of reports of peripheral neuropathy associated with atezolizumab compared to other ICIs, both in monotherapy and in combination therapy with ipilimumab/nivolumab. Although some case reports of neuropathy following atezolizumab treatments were reported in the literature (40–42), these differences in neurological adverse events reporting with anti-PD-L1 agents, in particular atezolizumab, compared to other ICIs have not been previously documented in the literature. Thus, the cause of this higher frequency of reporting remains largely unknown, requiring further investigation.

## 5 Study strengths and limitations

Our study has inherent limitations due to the post-marketing surveillance system, as it was based on data from the European pharmacovigilance reporting system. For example, considering that safety reports are mainly sent in a spontaneous way by patients and physicians, they may be affected by the so-called underreporting phenomenon (43). In addition, the data reported in spontaneous ICSRs may often be incomplete (44). Moreover, information about the patients’ previous predisposing conditions is not available. These can only be inferred from the reported concomitant and suspected medications. As we did not have full-level access to EV, we were unable to analyze the time to onset or time to resolution, or the median age of the patients, which was only reported as an age group. Despite these limitations, pharmacovigilance databases are useful tools for monitoring the safety of medicines and represent a significant source of information (44). Spontaneous reporting systems allow for a better characterization of the safety profile of drugs and overcome the inherent limitations of clinical trials (45, 46). In recent years, the efforts of regulatory agencies and the scientific community have greatly improved the quantity, quality, and timeliness of ADRs reporting. These advances have increased the value of spontaneous reporting systems for post-marketing pharmacovigilance purposes. It is worth emphasizing that drug and vaccine safety signals require further *ad hoc* confirmatory investigations based on different study designs in order to validate them and evaluate the hypothetically necessary regulatory actions required (47, 48).

## 6 Conclusion

PNs are one of the most frequent ICIs-induced neurological ADRs. We analyzed data from the European pharmacovigilance database on the association between ICIs and PNs. In our study, we found a total of 766 PNs in 735 patients treated with immunotherapy. These included GBS, Miller-Fisher syndrome, carpal tunnel syndrome, neuritis, and demyelinating polyneuropathy. These ADRs were often serious, resulting in disability or hospitalization for the patient. Given the negative outcomes associated with the reported PNs, preventing their occurrence and obtaining earlier treatment would certainly improve the quality of life of patients with a better chance of a complete recovery and also reduce the associated healthcare costs. Moreover, our disproportionality analysis revealed an increased frequency of reported PNs after treatment with atezolizumab compared to other ICIs. This result requires further investigation to better characterize this potential risk. In this context, continuous monitoring of the safety profile of ICIs in real-life settings and conducting pharmacovigilance studies serve as essential instruments to identify specific safety signals. Tools to evaluate and monitor the safety of these new therapeutic approaches in clinical use are crucial to improving public health and reducing costs to healthcare systems. Pharmacovigilance remains a key component of effective public health programs aimed at ensuring the safer use of medical interventions for patients. In this context, the description and analysis of irADRs collected in pharmacovigilance datasets can increase the knowledge of possible ICI-related ADRs, making them more easily identifiable and better managed by clinicians and oncologists.

Finally, advances in immunotherapy have greatly and positively changed the way cancer is managed and controlled. However, despite all their benefits, scientific evidence demonstrates that irADRs related to ICIs are a common cause of disability in oncology patients and require continuous monitoring.

## Data availability statement

The datasets presented in this study can be found in online repositories. The names of the repository/repositories and accession number(s) can be found below: www.adrreports.eu.

## Author contributions

Conceptualization, AC and RD; AC and FR designed the study and managed the project; RR, FF, RDN, and NB drafted the manuscript; methodology, AC and RR; data curation, MT, NB, FF, RDN, and RR; writing—original draft preparation, RR and FF; RDN, and NB prepared the figures; writing—review and editing, CP and CR; supervision, MR and FR; project administration, MR and RD; funding acquisition, RD. All authors contributed to the article and approved the submitted version.
