# Supplementary material for: Do peripheral neuropathies differ among immune checkpoint inhibitors? Reports from the European post-marketing surveillance database in the past 10 years
Source: Front Immunol. 2023 Mar 16;14:1134436. doi: 10.3389/fimmu.2023.1134436 (PMC10060793; doi:10.3389/fimmu.2023.1134436)
Supplement: Supplementary file 1 [file Table_1.docx]

***Supplementary materials.***

**Table S1. Demographic characteristics and distribution for a primary source, country of primary source for regulatory purposes, number of suspected drugs other than immune checkpoint inhibitors (ICIs), and number of concomitant drugs of Individual Case Safety Reports (ICSRs) reporting at least one peripheral nerve adverse event and having one, two or more ICIs as suspected drug among those reported in the Eudravigilance database from the date of marketing authorization to 07/02/2020.**

| **Variable** | **Level** | **ICSRs**  **with**  **avelumab**  **(N=2; 0.3% )** | **ICSRs**  **with**  **cemiplimab**  **(N=1; 0.1%)** | **ICSRs**  **with**  **nivolumab, ipilimumab, and pembrolizumab**  **(N=5; 0.7%)** | **ICSRs**  **with**  **ipilimumab and pembrolizumab**  **(N=4; 0.6%)** |
| --- | --- | --- | --- | --- | --- |
| ***Age Group*** | *Adult* | - | - | 2 (40.0) | 1 (25.0) |
|  | *Elderly* | 2 (100) | 1 (100) | 3 (60.0) | - |
|  | *Not specified* | - | - | - | 3 (75.0) |
| ***Gender*** | *W (%)* | 1 (50.0) | - | 2 (40.0) | 3 (75.0) |
|  | *M (%)* | 1 (50.0) | 1 (100) | 3 (60.0) | 1 (25.0) |
|  | *Missing (%)* | - | - | - | - |
| ***Primary Source*** | *Healthcare Professional* | 2 (100.0) | 1 (100) | 5 (100) | 4 (100) |
|  | *Non-Healthcare Professional* | - | - | - | - |
|  | *Not available* | - | - | - | - |
| ***Primary Source Country for Regulatory Purposes*** | *European Economic Area* | 1 (50.0) | - | 2 (40.0) | 1 (25.0) |
|  | *Non-European Economic Area* | 1 (50.0) | 1 (100) | 3 (60.0) | 3 (75.0) |
|  | *Not available* | - | - | - | - |
| ***Suspected drug(s) other than ICIs*** | *0* | 1 (50.0) | 1 (100) | 4 (80.0) | 4 (100) |
|  | *1* | - | - | - | - |
|  | *2* | - | - | 1 (20.0) | - |
|  | *3* | 1 (50.0) | - | - | - |
|  | *4* | - | - | - | - |
|  | ≥ 5 | - | - | - | - |
| ***Concomitant drug(s)*** | *0* | - | 1 (100) | 2 (40.0) | 2 (50.0) |
|  | *1* | - | - | - | 1 (25.0) |
|  | *2* | 2 (100) | - | - | - |
|  | *3* | - | - | 1 (20.0) | - |
|  | *4* | - | - | - | 1 (25.0) |
|  | ≥ 5 | - | - | 2 (40.0) | - |

**Table S2. Distribution of neurological complications included in the “Peripheral neuropathies” HGLT that occurred in European patients treated with at least one ICI. Neurological complications were categorized according to MedDRA High-Level Terms (HLT) and the ICI treatments involved.**

| **High-Level Terms** | **Cemiplimab (N=1)** | **Avelumab (N=3)** | **Other ICIs combinations (N=10)** |
| --- | --- | --- | --- |
| **Peripheral neuropathies (NEC)** | **1 (100)** | **3 (100)** | **4 (40.0)** |
| Peripheral Neuropathy | - | 2 (66.7) | 3 (30.0) |
| Polyneuropathy | 1 (100) | - | 1 (10.0) |
| Peripheral sensory neuropathy | - | - | - |
| Autoimmune neuropathy | - | - | - |
| Peripheral motor neuropathy | - | - | - |
| Axonal neuropathy | - | 1 (33.3) | - |
| Peripheral sensorimotor neuropathy | - | - | - |
| Brachial plexopathy | - | - | - |
| Immune-mediated neuropathy | - | - | - |
| Toxic neuropathy | - | - | - |
| Neuralgic amyotrophy | - | - | - |
| **Acute polyneuropathies** | **-** | **-** | **5 (50.0)** |
| Guillain-Barré syndrome | - | - | 4 (40.0) |
| Acute polyneuropathy | - | - | 1 (10.0) |
| Acute motor axonal neuropathy | - | - | - |
| Acute motor-sensory axonal neuropathy | - | - | - |
| **Chronic polyneuropathies** | **-** | **-** | **1 (10.0)** |
| Demyelinating polyneuropathy | - | - | - |
| Chronic inflammatory demyelinating polyradiculoneuropathy | - | - | 1 (10.0) |
| Diabetic neuropathy | - | - | - |
| Multifocal motor neuropathy | - | - | - |
| Polyneuropathy in malignant diseases | - | - | - |
| **Mononeuropathies** | **-** | **-** | - |
| Carpal tunnel syndrome | - | - | - |
| Mononeuropathy multiplex | - | - | - |
| Peroneal nerve palsy | - | - | - |
| Phrenic nerve paralysis | - | - | - |
| Mononeuritis | - | - | - |
| Mononeuropathy | - | - | - |
| Peripheral nerve lesion | - | - | - |
| Peripheral nerve palsy | - | - | - |
| Radial nerve palsy | - | - | - |
| Sciatic nerve neuropathy | - | - | - |
| **Peripheral neuropathies** | **-** | - | - |
| Neuritis | - | - | - |
| **Acute Peripheral neuropathies** | **-** | - | - |
| Miller-Fisher syndrome | - | - | - |
